# *Capparis spinosa L.* aqueous extract evokes antidiabetic effect in streptozotocin-induced diabetic mice

**Published:** 2017

**Authors:** Mohamed Eddouks, Ahmed Lemhadri, Morad Hebi, Ahmed EL Hidani, Naoufel Ali Zeggwagh, Bachir EL Bouhali, Lhoussaine Hajji, Remy Burcelin

**Affiliations:** 1 *Faculty of Sciences and Tecniques Errachidia, Moulay Ismail University, BP 509, Boutalamine, Errachidia, 52000, Morocco*; 2 *UMR 5018 CNRS-UPS and IFR 31, Rangueil Hospital, L1 Bldg, BP 84225 Toulouse 31432 Cedex 4, France*; 3 *ISPITS Rabat, Avenue Hassan II Km 4,5 Route de Casa, Rabat Maroc*

**Keywords:** Diabetes, Streptozotocin, Insulin sensitivity, Mice

## Abstract

**Objective:**

As the aqueous extract of *Capparis spinosa* (CS) possess antidiabetic effect, he present study aims to reveal the possible mechanism of action of CS in diabetic mice.

**Materials and Methods:**

Both single and repeated oral administrations of aqueous extract of CS were performed in multi-low dose streptozotocin-induced (MLDS) diabetic mice. Euglycemic hyperinsulinemic clamp was used in association with the endogenous glucose production (perfusion rate of 3-^3^H glucose) to evaluate the effect of CS aqueous extract on insulin sensitivity.

**Results:**

Our study showed that aqueous extract of CS possess a potent hypoglycaemic activity in MLDS diabetic mice. Furthermore, the analysis perfusion of 3-^3^H glucose demonstrated the parallel decrease of basal endogenous glucose production (EGP) with the hypoglycaemic activity. EGP was lower in CS-Treated group when compared to the control group (p<0.001). The euglycemic hyperinsulinemic clamp technique demonstrated that CS treatment improves insulin sensitivity in peripheral tissues.

**Conclusion:**

We conclude that the antihyperglycemic effet CS is probably due to the inhibition of basal endogenous glucose production and the improvement of insulin sensitivity in MLDS diabetic mice.

## Introduction

Diabetes mellitus is a chronic disease characterized by hyperglycaemia resulting from the defects in either insulin secretion or insulin action (American Diabetes Association, 2014 ▶). In both type 1 and 2 diabetes mellitus, hyperglycemia, insulin resistance have been observed in addition to various physiological complications. Insulin resistance is a pathophysiological abnormality generally associated with hypertension, dyslipidemia, central obesity and coronary heart failure (Karamanou et al., 2016 ▶). Insulin resistance is clinically defined as the inability of exogenous or endogenous insulin to inhibit endogenous glucose production or to increase glucose uptake and utilisation in normal subjects (Lebovitz, 2001 ▶). Insulin-signalling pathway alteration seems to be the main cause of the insulin-resistant state (Karamanou et al., 2016 ▶). 

Stable glucose homeostasis is generally associated with the ability of insulin to mediate tissue glucose uptake. Plants and herbs have been used in folk medicine for their medicinal and protective properties. Recent studies have confirmed the insulin sensitivity improvement of many medicinal plants and herbal preparations (Ghorbani, 2013a; Ghorbani, 2013b). *Capparis spinosa* L. (CS) (Capparidaceae) was reported to have a number of potentially pharmacological activities including anti-inflammatory, anti-allergic, antidiabetic, hypolipidemic, hepatoprotective, antimicrobial, antiviral, immunomodulatory, antioxidant, anti-apoptotic, melanogenesis stimulating, antimutagenic, antiparasitic, antihypertensive, antiproliferative, antifungal, anti-HIV, anti-Helicobacter pylori, anti-complement and cardioprotective effects (Eddouks, 2015 ▶; Mousavi et al., 2015). Ethnopharmacological survey showed that CS is traditionally used in diabetes control and treatment in Morocco (Eddouks et al., 2002). Furthermore, we have reported the hypoglycaemic and hypolipidemic activities of the aqueous extract of CS fruits in streptozotocin-induced diabetic rats, an animal model of human type 1 diabetes mellitus (Kazemian et al., 2015, Eddouks et al., 2004 ▶; Eddouks et al., 2005 ▶; Lemhadri et al., 2007 ▶). We have demonstrated the potent hypolipidemic and anti-obesity effects of caper in high-fat diet fed mice (Lemhadri et al., 2007 ▶). Recently, a controlled human study has showed that CS possess hypoglycaemic effect in Type 2 diabetic patients (Huseini et al., 2013 ▶). However, the mechanism of action underlying the antidiabetic effect of CS is not yet established. Therefore, the present study aims to investigate the potential hypoglycaemic activity of aqueous extract of CS in multi-low dose streptozotocin-induced (MLDS) diabetic mice, another type 1 diabetes animal model and to evaluate the possible beneficial effects of CS fruits on hepatic glucose production and peripheral insulin sensitivity. The euglycemic clamp technique is considered as the reference tool for assessing insulin resistance and insulin sensitivity was used (Eddouks et al., 2012 ▶). Endogenous glucose production change was estimated in MLDS diabetic mice using primed-continuous 3-^3^H glucose perfusion.

## Materials and Methods

**Plant material**

Fruits of *Capparis spinosa* (Capparidaceae) were collected from the Tafilalet region of Morocco and air-dried at 40°C. Specimen (ME 60) was deposited at the herbarium of the Faculty of Sciences and Techniques Errachidia.

**Preparation of the aqueous extract**

Plant material was prepared according to the traditional method used in Morocco (decoction): 1 g of powdered fruits mixed with 100 ml distilled water was boiled for 10 mins and then cooled for 15 min. Thereafter, the aqueous extract was filtered using a Millipore filter (Millipore 0.2 mm, St-Quentin-en-Yvelines, France) to remove particulate matter. The filtrate was then freeze-dried and the desired dose (mg of lyophilized aqueous extract of CS fruits per kg body weight) was then prepared and reconstituted in 1.5 ml of distilled water. The extracts obtained were then given orally to different groups of mice at the dose of 20 mg/kg body weight. The extract was maroon colored with a percentage yield between 10 and 15%. Its average osmolarity was 50 mOsm, pH 6.1, and it had a very low viscosity. 

**Experimental animals**

Eleven-week-old male C57BL/6J mice weighing 30±5g were used in all experiments under under controlled environment (inverted 12-h daylight cycle, lights off at 10 am) at 22°C with free access to food and water in groups of five mice per cage. All animal experimental procedures have been approved by the local ethical committee of the Rangueil hospital (Toulouse, France). 

**Induction of diabetes**

Four days consecutive repeated intraperitoneal injection of streptozotocin (80 mg/kg) was performed in fasted adult male C57BL/6J mice in order to induce diabetes. Daily injections were repeated at the same time (10.00 am). Streptozotocin (STZ) (Sigma, St Louis, Mo, USA) was extemporaneously prepared in 0.1 M fresh cold citrate buffer at pH 4.5. Mice with blood glucose levels higher than 16 mM were considered as diabetic and then included in this study. 

**Acute and chronic oral treatment**

Normal and diabetic mice were randomly assigned to two groups (n= 6 in each group). The control group received distilled water and the treated group received aqueous extract of CS at the dose of 20 mg/kg B.W. This dose was chosen according the traditional use of this plant in Tafilalet region. The aqueous extract of CS and vehicle were administered orally by gavage once daily at 10 am. The hypoglycaemic effect was evaluated in fasted mice 1, 2, 4 and 6 hours after a single oral administration and after 4, 7 and 15 days of once daily repeated oral administration. 

**Surgery**

Intravenous perfusions was performed using a catheter implanted under anaesthesia with pentobarbital 1% into the femoral vein of male C57BL/6J mice (n=6 for each experimental group). The other extremity of the catheter exteriorized, and secured at the back of the neck. The mice were then allowed to recover from the surgery in individual cages. 

**Endogenous glucose production**

Control group received continuous perfusion of physiological saline solution (NaCl 0.9%) and experimental group received continuous perfusion of aqueous extract of CS (20 mg/kg) at a rate of 2 μl/min/kg body weight were used. The mice were fasted for six hours prior to the infusions. They were connected to the infusion apparatus two hours prior to the start of the infusions with free access to water. In order to determine the rate of endogenous glucose production (EGP) in mice at post-absorptive state, 3-^3^H-glucose (Perkin Elmer, Boston, MA) was infused continuously at a dose of 30 Ci/kg/min to ensure a detectable plasma D-(3H)^3^-glucose enrichment. Plasma glucose and 3-^3^H-glucose were determined in 5 l of blood sampled from the tip of the tail vein each 20 min following the 3-^3^H-glucose or saline infusion. 

**Euglycemic-hyperinsulinemic glucose clamp**

The animals were allowed to recover from surgery for four days. Insulin sensitivity was assessed by using euglycemic hyperinsulinemic clamp as described previously (Burcelin et al., 2002 ▶). Briefly, 6-hr–fasted mice were divided randomly into two groups (n=6 for each group). The control group was continuously infused with insulin at a rate of 4 mU/kg^/^min (physiological) and saline solution (2 μl/min) for 3 hr. In addition to insulin infusion, the experimental group was infused with aqueous extract of CS (20 mg/kg) at a rate of 2 μl/min. Throughout the infusion, blood glucose levels were assessed from blood samples (5 μl) collected from the tip of the tail vein when needed using a blood glucose meter (Roche Diagnostic, Meylan, France). Euglycemia was maintained at 5 mM by periodically adjusting a variable infusion of 16.5% glucose. Plasma glucose concentrations were determined in 5 μl of blood samples collected from the tip of the tail vein every 10 min during the last hour of the infusion. 

**Calculations**

3-^3^H-glucose enrichment was determined from the total blood after deproteinization which was performed as follows: 5 l of venous blood was mixed with 250 l of 0.3M Ba(OH)_2_ which was added to precipitate the proteins and blood cells. The Zn(OH)_2_ precipitate formed was spun down (Burcelin et al., 1995 ▶). An aliquot of the supernatant was evaporated to dryness to determine the radioactivity corresponding to 3-^3^H-glucose. Another aliquot was directly mixed with the scintillation buffer to determine the radioactivity corresponding to 3-^3^H-glucose. Thereafter, the difference between the first and the second aliquot corresponds to ^3^H produced. In the third aliquot of the same supernatant, glucose concentration was assessed by the glucose oxidase method (Trinder, St Louis, Mo). Calculations were done with parameters collected during the last 60 min of the infusions when a steady-state 3-^3^H-glucose enrichment was obtained. Glucose turnover was calculated by dividing the 3-^3^H-glucose infusion rate by the plasma glucose specific activity. A steady specific activity with variations smaller than 15% was obtained during the last hour of the infusion. Mice showing larger variations in the specific activity were excluded from the study.

The total amount of the infused glucose was considered to be taken up by the body tissues and, under these steady-state conditions of euglycemia and hyperinsulinemia, glucose input was equal to glucose utilisation. The glucose infusion rate (GIR) was calculated every 10 min during the clamp study. Metabolic clearance rate of glucose (MCR, mg/kg/min) was then obtained from GIR divided by the corresponding blood glucose concentration. 

**Statistical analysis**

Data were expressed as mean ± SEM. Statistical differences among the means were assessed by two-way ANOVA followed by Bonferroni multiple comparisons test with GraphPad Prism 6 software. Differences were considered to be significant when p<0.05.

## Results

**Blood glucose levels**

**Figure 1:**
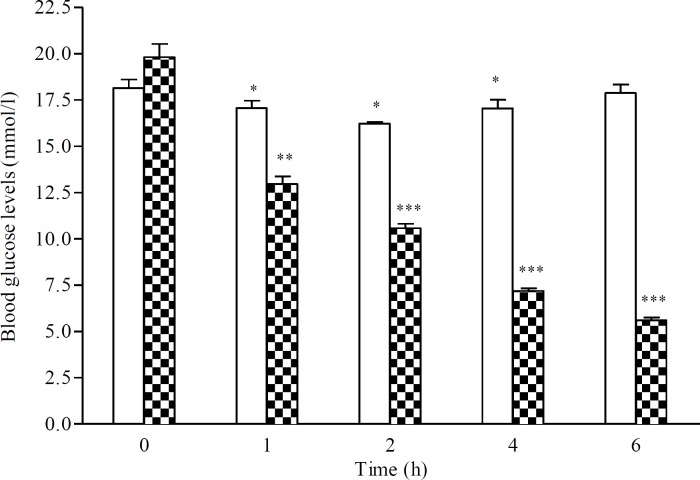
Plasma glucose levels (mM) over 6 hr after single oral administration of CS aqueous extract (20 mg/kg) in diabetic mice. Data are expressed as means   S.E.M. of n= 6 mice per group. *p<0.05, and **p<0.01 and ***p<0.001 when as to baseline values (hr 0). : Control, : *Capparis spinosa*

*Single oral administration:* Figure 1 depicts the blood glucose levels lowering effect of single oral administration of the aqueous extract of CS fruits (20 mg/kg) in MLDS diabetic mice. In control diabetic rats, the blood glucose levels were decreased between the first and the fourth hour after the start of experiment perhaps due to long post absorptive state. CS treatment was accompanied with a more important decrease in blood glucose levels when compared with the control group. Blood glucose levels dropped from 19.81±1.61 to 10.57±0.54 and 5.59±0.36 mM two (p<0.001) and six (p<0.001) hours after treatment, respectively (Figure 1). 

**Repeated oral administration**

**Figure 2:**
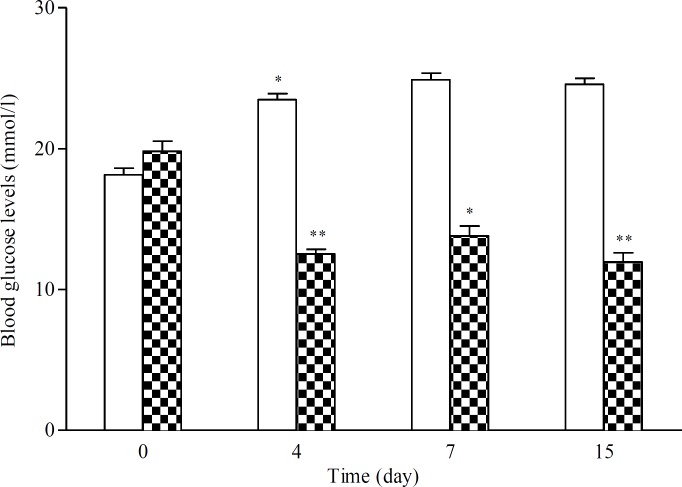
Plasma glucose levels (mM) over once daily repeated oral administration of aqueous extract of CS (20 mg/kg) for 15 days in diabetic mice. Data are expressed as means   SEM of n= 6 mice per group. *p<0.05 and **p<0.01 as compared to baseline values (Day 0). □: Control, ▓: *Capparis spinosa*

 The effect of once daily repeated administration of aqueous extract of CS (20 mg/kg) in MLDS diabetic mice is shown in Figure 2. In diabetic mice treated with CS extract, blood glucose levels were significantly decreased by 39%. It dropped from 19.81±1.61 to 11.96±1.43 mM at the end of treatment (p<0.01) (Figure 2). 

**Endogenous glucose production**

**Figure 3:**
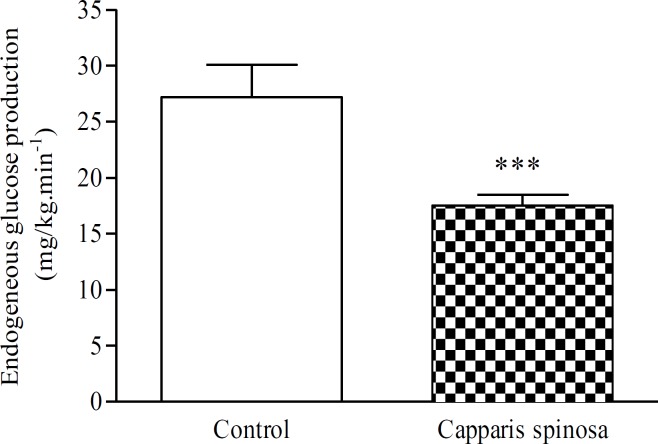
Endogenous glucose production (mg/kg/min) after the aqueous extract of CS infusion (20 mg/kg) in diabetic mice. Data are expressed as means   S.E.M. of n= 6 mice per group. ***p<0.001 as compared to control group

In MLDS mice, aqueous extract of CS infusion at the dose of 20 mg/kg produced a strong hypoglycaemic effect. Blood glucose levels were decreased from 18.83±0.44 to 6.66±1.44 mM, 3 hr after CS infusion (p<0.001) (Table 1). Parallel to the marked decrease in blood glucose levels, CS infusion (20 mg/kg) produced a strong decrease in endogenous glucose production (EGP). At the end of the infusion period, EGP values were significantly lower in CS-treated group as compared to the control one, 17.5±2.4 *vs* 27.2±7.1 mg/kg/min (p<0.001) (Figure 3).

**Metabolic clearance rate of glucose**

There are no significant differences in basal metabolic clearance rate (MCR) of glucose between CS-treated diabetic group and control group (Table 1).

**Table:** 

**Experimental** ** groups**	**Blood glucose levels (mM)**		**Metabolic clearance rate of glucose (mg/kg.min** ^-1^ **)**
	0h	3h	
**Control**	18.44±1.33	9.83±1.63 ***	0.0270±0.007
**CS-treated**	18.83±0.44	6.66±1.44 ***	0.0200±0.002 ^NS^

**Euglycemic hyperinsulinemic clamp**

**Figure 4.**
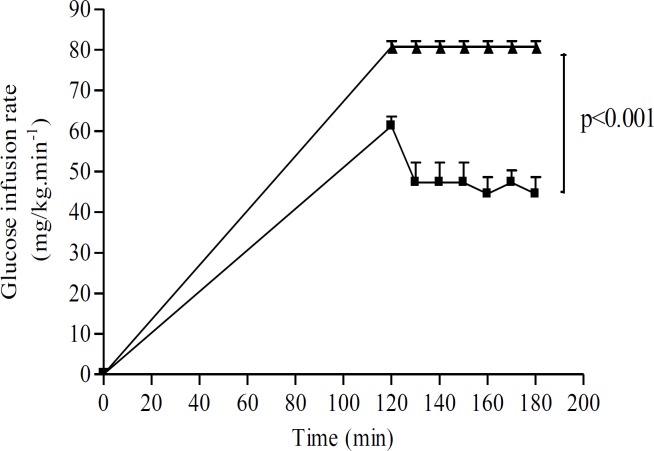
Glucose infusion rate (mg/kg/min) after aqueous extract of CS infusion (20 mg/kg) in diabetic mice. Data are expressed as means   SEM of n= 6 mice per group

Figure 4 depicts the time-course of glucose infusion rate (GIR) in CS-treated and control diabetic groups. The total amount of the infused glucose was considered to be taken up by the body tissues and, under these steady-state conditions of euglycemia and hyperinsulinemia, glucose input was equal to glucose utilisation. Because a GIR plateau was achieved during the last 30 min of the clamp procedure, GIR was used as an indicator of whole body glucose utilisation. So, GIR reflects the whole body insulin sensitivity. GIR was significantly higher in CS-treated group than in control group (80.72±3.47 *vs* 44.55±10.06 mg/kg/min respectively (p<0.001)) (Figure 4).

## Discussion

This study was designed to investigate the hypoglycaemic activity of the aqueous extract of CS and to evaluate the possible beneficial effect of CS infusion on endogenous basal glucose production and whole body insulin sensitivity in multi-low dose streptozotocin-induced diabetic mice. Multiple injection of STZ is known to affect β-cells, eliciting a subsequent immune destruction leading to β-cell lysis (Thorvaldson et al., 2003 ▶). This animal model is known to mimic some pathophysiological aspects of type 1 diabetes in human patients (Eddouks et al., 2012 ▶). Moreover, STZ injection induced alteration of insulin sensitivity one day after STZ injection and the insulin suppression of hepatic glucose output was reported to be impaired from the third day after STZ injection (Zhou et al., 2010 ▶). Furthermore, it has been reported that intracellular signal transduction, glucose transport system, and glucose metabolism accompanied the insulinopenic state induced by STZ (Zhou et al., 2010 ▶). Thus, these studies clearly linked impairments in post-binding mechanisms in the liver and skeletal muscles with the defective insulin action in STZ diabetic rats. The results clearly demonstrate that both single and repeated oral administration of aqueous extract of CS exert significant reduction in blood glucose levels in MLDS diabetic mice. This finding is in accordance with previous studies performed in STZ-induced diabetic rats (Kazemian et al., 20015; Eddouks et al., 2004 ▶), High fat diet mice (Lemhadri et al., 2007 ▶) and in type 2 diabetic patients (Huseini et al., 2013 ▶). A similar finding was reported in diabetic rats when treated with CS fruits extracts at the doses of 200 and 800 mg/kg for 28 days (Rahmani et al., 2013 ▶). Moreover, hydro-alcoholic extract of caper decreased blood glucose levels in alloxan-induced diabetic rats without affecting the pancreas (Hashemnia et al., 2012 ▶). However, the mechanism of action by which CS exerts its anti-diabetic effect is not determined. In this study, the effects of aqueous extract of CS on blood glucose levels, basal endogenous glucose production and insulin sensitivity were studied in awake and unrestrained diabetic mice. The results demonstrated that continuous infusion of CS extract for three hours significantly reduced both blood glucose levels and basal endogenous glucose production. However the metabolic clearance rate of glucose remains unchanged for unknown reasons. Similar findings have been shown with other plants extracts (Chen et al., 2014; Adeneye, 2012 ▶; Eddouks et al., 2003 ▶; Koyama et al., 2004 ▶). The liver plays a crucial role in the maintenance of blood glucose levels, and excessive hepatic glucose production is the main indicator of untreated or poorly controlled diabetes mellitus (Zhou et al., 2010 ▶). Glucogenolysis and gluconeoneogenesis are the main sources of liver glucose production. Excessive hepatic glucose production is a result of the lack of insulin repressing the two key gluconeogenic enzymes, phosphoenolpyruvate carboxykinase and glucose-6-phosphatase (Zhou et al., 2010 ▶). Inhibition of the activities of these two enzymes is the probable target of CS aqueous extract. Furthermore, diabetes mellitus is well characterized by the depletion in hepatic and muscular glycogen (Eddouks et al., 2014 ▶). So, it is not excluded that CS may also stimulate hepatic glycogenesis leading to reduce hepatic glucose production. Using the euglycemic hyperinsulinemic glucose clamp technique, this study clearly established that aqueous extract of CS infusion caused a major improved the whole body insulin sensitivity in MLDS diabetic mice. It is well known that hyperglycemia observed in STZ-induced diabetes leads to the early apparition of insulin resistance in hepatic and peripheral tissues (Zhou et al., 2010 ▶). In diabetic mice, during the insulin infusion rate at 4 mU/kg/min used in this study, insulinemia reached approximately the levels of 100 μU/ml (Tominaga et al., 1995 ▶; Eddouks et al; 2003 ▶). At high physiological insulin levels, endogenous glucose production can be completely suppressed under an insulin infusion rate of 3 mU/kg/min (Tominaga et al.1995 ▶). Therefore, the GIR is a valid indicator of the total body glucose utilisation. Consequently, our results suggest that CS administration would improve insulin sensitivity in peripheral tissues. Other herbal preparations and medicinal plants have been reported to improve insulin sensitizing in similar pathway (Manukumar et al., 2016 ▶). Peripheral insulin sensitivity enhancement may be a consequence of the stimulation of glucose transport and metabolism, insulin receptor phosphorylation or intracellular insulin signalling pathways (Eddouks et al., 2014 ▶; Hu et al., 2010 ▶). The precise mechanisms related to the insulin sensitizing property of aqueous extract of CS need further biochemical and molecular investigations .

In conclusion, this study demonstrates that CS treatment exhibited a hypoglycaemic effect which seems to be mediated through a decrease of endogenous glucose production and improvement of insulin sensitivity in multi-low dose STZ-induced diabetic mice. Our results give a major support of reported anti-hyperglycemic activity of *Capparis spinosa *extract that may be of importance in the treatment of insulin resistance.
